# Associations of muscle coactivation patterns with gait, muscle strength, and symptoms across different stages of knee osteoarthritis

**DOI:** 10.1186/s43019-026-00326-4

**Published:** 2026-07-06

**Authors:** lihang zhang, peiyao liang, hang yu, shaochen qu, liming liu, liufeng xiao, shiyi tong, dianwei li, shibo xu, xing xing, lin guo

**Affiliations:** 1 State Key Laboratory of Trauma, Burn and Combined Injury, Department of Orthopedics/Sports Medicine Center, First Affiliated Hospital of Army Medical University, Chongqing, China; 2 Department of Orthopedics of Jiangbei Campus, The First Affiliated Hospital of Army Medical University, Chongqing, China; 3 Department of Traditional Chinese Medicine Rehabilitation, Jiangbei Campus, First Affiliated Hospital of Army Medical University, Chongqing, China; 4 School of Public Health, Peking University, Beijing, China

**Keywords:** Knee osteoarthritis, Muscle activation, Gait analysis, Muscle strength, Electromyography, Biomechanics, Neuromodulation

## Abstract

**Objective:**

This study aimed to investigate associations between neuromuscular activation patterns, symptoms, muscle strength, and gait parameters across different stages of knee osteoarthritis (KOA).

**Methods:**

In this cross-sectional study, 165 unilateral KOA patients and 32 healthy controls underwent synchronized gait analysis, isokinetic strength testing, surface electromyography, and completed the Western Ontario and McMaster Universities Osteoarthritis Index (WOMAC) questionnaire. Muscle coactivation was quantified as the normalized electromyographic (EMG) ratio of antagonist to agonist muscles during gait phases and isokinetic contractions. Group comparisons were performed using one-way ANOVA with Bonferroni correction. Spearman correlations and multiple linear regression models examined relationships between muscle activation, biomechanical parameters, and WOMAC scores.

**Results:**

Compared with controls, patients with KOA showed progressively deteriorating gait patterns and strength performance with increasing K-L grades (*p* < 0.05). A distinct compensatory neuromuscular pattern, characterized by excessive coactivation of the hamstrings (particularly the lateral hamstring) and the vastus lateralis, was identified (*p* = 0.002–0.048). This pattern was significantly associated with reduced knee flexion moment and elevated knee adduction moment (*p* = 0.004–0.038). These changes were observable from K-L II and became more pronounced in advanced KOA (K-L ≥ III), correlating with higher WOMAC pain, stiffness, and functional scores (*p* < 0.001–0.046). Significant inter-limb asymmetry was observed between affected and contralateral limbs (asymmetry index [ASI] = 1.72–13.84; limb symmetry index [LSI] = 56.3–92.7%).

**Conclusions:**

KOA is associated with an adaptive neuromuscular strategy characterized by excessive hamstring coactivation. While this pattern may aim to stabilize the joint, it correlates with biomechanical inefficiency, symptom worsening, and functional decline. This coactivation pattern may serve as a promising biomechanical marker for better understanding KOA manifestations and warrants further investigation as a potential therapeutic target.

**Supplementary Information:**

The online version contains supplementary material available at 10.1186/s43019-026-00326-4.

## Introduction

Knee osteoarthritis (KOA) involves chronic pain, disability, and muscle weakness [1]. Quadriceps weakness, stemming from both atrophy and neural activation deficits, impairs lower limb function [2]. Since activation deficits may occur early and influence disease progression, their detection is crucial [3, 4]. As KOA advances, declining quadriceps activation is often accompanied by increased hamstring coactivation [5]. Investigating this evolving balance between muscle activation and coactivation across disease stages may inform targeted exercise prescriptions to better hinder progression.

Reduced knee flexion during stance is a hallmark gait alteration in KOA, potentially stemming from quadriceps weakness [6–9]. This diminished flexion compromises the joint’s shock-absorbing capacity, increasing vertical loading and potentially accelerating disease progression [3]. Quadriceps activation deficits, which can occur early in the disease, impair force generation and contribute to weakness, creating a worsening cycle [10, 11]. Muscle coactivation, the simultaneous activation of agonist and antagonist muscles, is a normal stabilizing mechanism during gait, primarily occurring in early stance and late swing [12, 13]. The hamstrings play a pivotal role in controlling knee flexion and preventing hyperextension [14]. However, in KOA, particularly in moderate to severe stages, hamstring coactivation becomes excessive [15–17]. Distinguishing between adaptive and maladaptive coactivation is essential, recognizing that they exist on a continuum rather than as a strict dichotomy. In early-stage KOA, moderate antagonist coactivation may play a compensatory role by enhancing joint stability and redistributing mechanical loads, a pattern considered adaptive [18–21]. With disease progression, however, coactivation frequently becomes excessive and persistent. This sustained elevation may compromise net joint moments, elevate joint loading, and exacerbate clinical symptoms, constituting maladaptive coactivation [22, 23]. We operationalize this distinction by analyzing associations between coactivation levels and functional outcomes, including gait efficiency, muscle strength, and patient-reported outcomes.

Previous studies on quadriceps alterations in KOA have focused on advanced stages (K-L III and IV), leaving early-stage changes poorly characterized [24, 25]. To clarify this progression, we enrolled patients across all K-L grades. We recorded surface electromyographic (sEMG) signals during walking to investigate gait-related muscle activation. Since gait biomechanics can be influenced by speed, pain, and obesity, which may obscure correlations with structural changes [26–28], we also recorded sEMG during standardized isokinetic strength testing. Muscle activation during controlled force assessment offers a reproducible and standardized means for evaluating neuromuscular control between individuals [12]. This dual-modality approach, assessing activation during both functional gait and controlled force generation, enables a more systematic evaluation of neuromuscular control across disease stages, which is helpful for developing effective early rehabilitation strategies. The primary objective of this study is to investigate the gait, muscle strength, muscle activation patterns, and patient-reported outcomes across different stages of KOA. The secondary aim is to analyze the associations between these biomechanical parameters and patient-reported outcomes. Finally, we sought to explore the relationships between muscle activation and both gait and strength parameters, thereby providing a more integrated understanding of neuromuscular control in KOA progression.

## Methods

### Trial design and participants

This cross-sectional study recruited a total of 197 participants from the outpatient department of the affiliated hospital and the community between April 2025 and December 2025. Among them, 165 participants had strictly unilateral radiographic and symptomatic knee osteoarthritis (KOA), and 32 healthy adults without radiographic or symptomatic KOA were recruited from the community as healthy controls. Unilateral KOA was defined as: (1) the affected knee exhibiting K-L grade I–IV on the basis of weight-bearing anteroposterior radiographs, accompanied by pain or functional symptoms for at least 6 months, and (2) the contralateral knee showing no radiographic evidence of KOA and no history of pain or functional limitation. The inclusion and exclusion criteria are detailed in the supplementary information [29, 30]. Each participant’s X-rays were independently reviewed by two senior orthopedists who were blinded to patients’ clinical status. If no consensus was reached on the K-L grade, a third senior orthopedist made the final adjudication (intra-observer ICC = 0.94; inter-observer ICC = 0.91). KOA patients were divided into four groups on the basis of the K-L criteria scale, including K-L I (*n* = 39), K-L II (*n* = 38), K-L III (*n* = 41), and K-L IV (*n* = 47). All participants signed written informed consent forms. The study adhered to the ethical principles of the Declaration of Helsinki and was approved by the local ethics review committee.

### Western Ontario and McMaster Universities Osteoarthritis Index (WOMAC)

The WOMAC index, developed in 1988, assesses pain and function in hip and knee osteoarthritis [31]. The scale consists of 24 questions, each evaluated as a 5-point Likert. The pain subscale is scored between 0 and 20. The stiffness subscale is scored between 0 and 8. And the function subscale possesses seventeen items and is scored between 0 and 68. A high score indicates poor function, pain, or stiffness [32]. For completeness, we also administered the WOMAC questionnaire to healthy controls; their scores were uniformly low (near floor), as expected, and the detailed data are provided in Table 1.

**Table 1:** Participant characteristics

Knee OA group (*n* = 165)									Control (*n* = 32)		*p*1	*p*2
	K-L I (*n* = 39)		K-L II (*n* = 38)		K-L III (*n* = 41)		K-L IV (*n* = 47)					
	Male (*n* = 18)	Female (*n* = 21)	Male (*n* = 18)	Female (*n* = 20)	Male (*n* = 17)	Female (*n* = 24)	Male (*n* = 21)	Female (*n* = 26)	Male (*n* = 13)	Female (*n* = 19)		
Age (years)	60.6 ± 7.1	59.3 ± 9.2	60.5 ± 9.3	59.4 ± 10.6	62.1 ± 7.1	60.1 ± 7.4	61.0 ± 9.4	61.5 ± 10.1	61.0 ± 8.8	60.3 ± 7.6	0.159	0.301
Height (cm)	170.3 ± 6.8^ɑ^	157.3 ± 5.3	168.6 ± 7.3^ɑ^	157.1 ± 7.1	169.8 ± 7.7^ɑ^	156.7 ± 6.1	167.9 ± 6.7^ɑ^	156.5 ± 4.3	170.6 ± 7.3^ɑ^	157.2 ± 4.5	0.463	0.707
Weight (kg)	69.7 ± 7.7^ɑ^	64.1 ± 8.1	68.1 ± 6.9^ɑ^	62.8 ± 7.3	68.7 ± 7.4^ɑ^	63.7 ± 7.1	67.9 ± 8.2^ɑ^	64.3 ± 6.6	68.8 ± 8.7^ɑ^	63.3 ± 7.7	0.765	0.941
BMI (kg/m^2^)	23.9 ± 3.8	25.3 ± 4.6	24.3 ± 3.1	25.0 ± 3.9	23.7 ± 3.6	25.6 ± 4.2	24.5 ± 4.0	25.2 ± 3.8	23.6 ± 3.5	24.2 ± 3.5	0.163	0.470
WOMAC–pain	7.31 ± 3.67*	7.74 ± 4.27*	8.83 ± 4.94*	9.03 ± 4.56*	9.87 ± 5.62^ɑ^*	12.98 ± 4.01*	11.01 ± 5.31^ɑ^*	15.43 ± 6.17*	0.31 ± 0.62	0.54 ± 0.82	< 0.001*	< 0.001*
WOMAC–stiffness	2.07 ± 1.67*	2.19 ± 1.94*	2.21 ± 2.07*	2.65 ± 1.67*	4.07 ± 2.18^ɑ^*	6.33 ± 2.76*	5.21 ± 3.06^ɑ^*	7.97 ± 3.27*	0.13 ± 0.34	0.23 ± 0.50	< 0.001*	< 0.001*
WOMAC–physical function	24.71 ± 11.60*	25.63 ± 12.88*	26.61 ± 13.17*	26.97 ± 12.63*	28.37 ± 14.26*	35.96 ± 13.72*	31.73 ± 13.88^ɑ^*	39.94 ± 13.65*	0.93 ± 1.20	0.72 ± 1.54	< 0.001*	< 0.001*

Data are presented as mean ± standard deviation. K-L, Kellgren–Lawrence grade; BMI, body mass index; WOMAC, Western Ontario and McMaster Universities Osteoarthritis Index. Statistical value refers to ANOVA or the independent Student’s *t*-test. The overall ANOVA *p*-values (*p*1 for males, *p*2 for females) indicate whether significant differences exist among the five groups. *Indicates significant differences between KOA groups and the control group after Bonferroni correction. ^ɑ^Indicates significant differences between male and female subjects within the same K-L grade (independent *t*-test, *p* < 0.05)

### Gait analysis

**Fig. 1:**
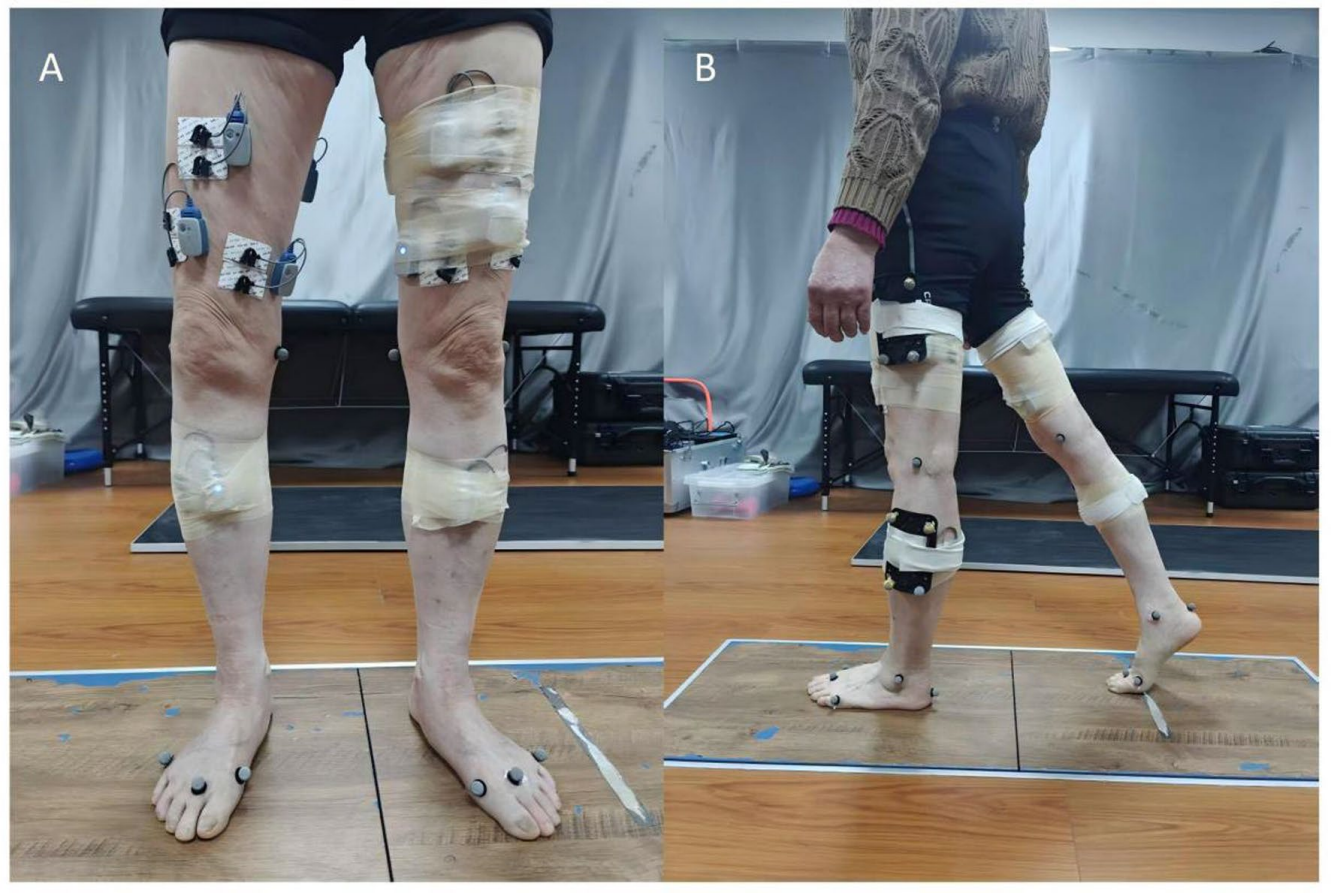
**a** The distribution of electromyography during the gait test process. **b** Gait test using combined electromyography

After familiarization with the protocol, participants walked barefoot at a self‑selected comfortable speed, defined as their natural walking pace without any external speed guidance. The average speed over three trials was calculated from motion capture data over the middle 6 m of the 12‑m walkway. Reflective markers were placed on the pelvis and lower limbs [33]. Data were collected using a six-channel Bertec force plate (AMTI, BMS-400600-2 K, USA, 1000 Hz) and a ten-camera motion capture system (Qualisys ArqusA5, Sweden, 100 Hz). Following a static standing trial, three walking trials were performed, and then the average of these was used for analysis (Fig. 1). Visual 3D™ software (C-Motion, Germantown, MD, USA) was used to obtain spatiotemporal parameters and kinematic and kinetic data [34–37]. In addition, the asymmetry index (ASI) and dynamic margins of stability (MoS) were also calculated. The former is used to assess the asymmetry of various parameters (formula 1) [38]. According to the method of Hof and Ramanujam et al. [38–41], dynamic MoS is used to assess the stability of limbs during dynamic activities (formula 2–4). The following are all the formulas that need to be used:

1 $$ASI=\frac{100*\left|{V}_{affected}-{V}_{contralateral}\right|}{0.5*\left|{V}_{affected}+{V}_{contralateral}\right|}$$

2 $$X_{CoM} = X + V/\omega$$

3 $$\upomega = \sqrt{\mathrm{g}/\mathrm{l}}$$

4 $$\mathrm{M}\mathrm{OS}=\mathrm{XCoM}-\mathrm{BoS}$$

### Measures of muscle strength

**Fig. 2:**
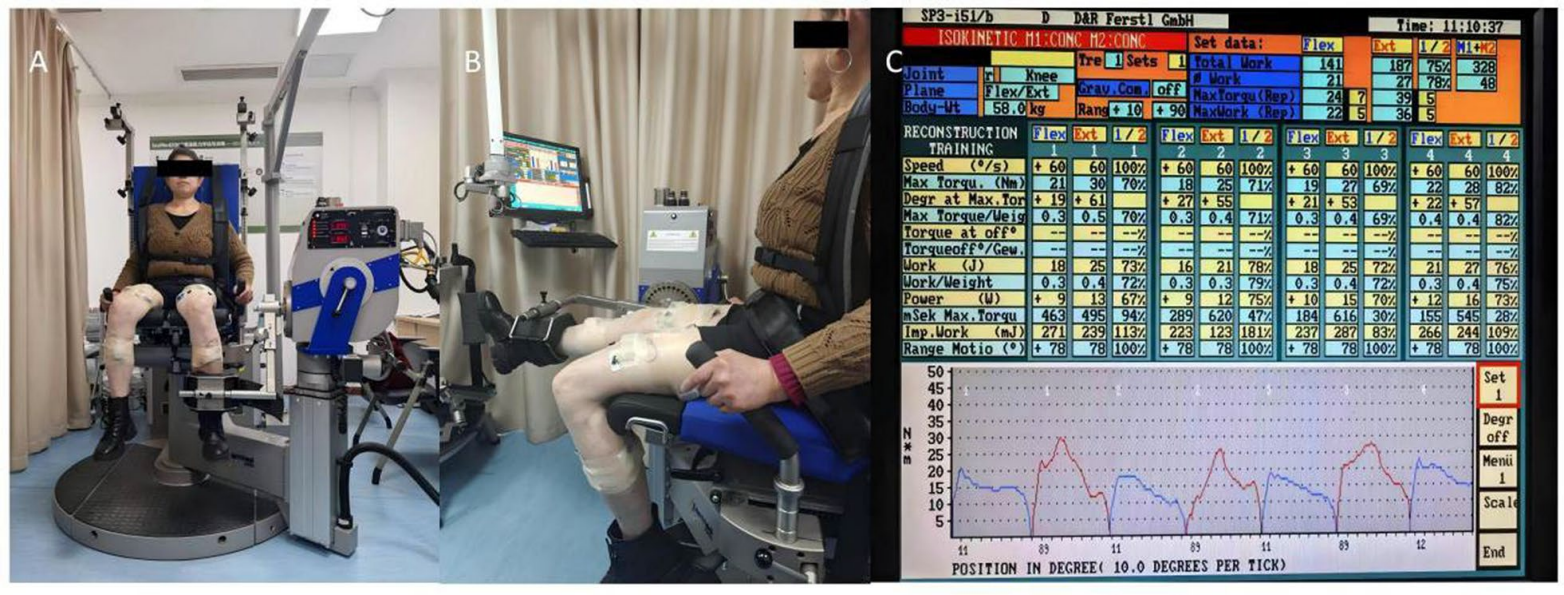
**a** Isokinetic muscle strength testing combined with electromyography recording. **b** The visual feedback system during the isokinetic muscle strength test. **c** The sample result of the isokinetic muscle strength test (the name of the subject has been obscured)

Participants completed isokinetic strength test using a dynamometer (Isomed 2000, D&R Ferstl GmbH, Germany) [42, 43]. The patients were instructed to sit with their hips and knees at 90°, the trunk, pelvis, and thighs were stabilized. The device’s laser locator was aligned with the knee’s flexion–extension axis for measurement. Following a warm-up with gravity correction, participants performed five maximal concentric knee flexion/extension repetitions at 60°/s, arms crossed (Fig. 2). Testing order was healthy limb first, then affected limb. All outcome measures were calculated using the software package provided by the manufacturer (IsoMedAnalyze). The results included average peak torque (APT), joint angle at peak torque (PTA), the time to peak torque (TPT), and average power (AP; total work divided by the time). The maximum strength of antagonists relative to the maximum strength of agonists is frequently considered as an index of muscle strength balance. For the knee joint, muscle balance was assessed via the concentric hamstrings/quadriceps (H/Q) ratio [44]. As previously described [45], the raw data were converted to torque (Nm), normalized to body weight (Nm/kg), and averaged to determine maximal isokinetic strength. Finally, the limb symmetry index (LSI) was also calculated for each patient. The following formula is used [46]:

LSI = (affected limb/contralateral limb) × 100.

Given that LSI may underestimate quadriceps weakness [47], strength was expressed using two dependent variables, namely LSI and peak torque normalized to body weight, as described by Kuenze et al. [48].

### Surface electromyography (sEMG)

sEMG was concurrently recorded during gait and isokinetic strength tests using a 16-channel wireless system (Noraxon Ultium, Noraxon, USA). Following the Surface Electromyography for Non-Invasive Assessment of Muscles (SENIAM) guidelines [29], the skin was prepared and electrodes were placed in a straight line aligned with the vastus medialis, vastus lateralis, and medial/lateral hamstrings to measure muscle activation. The differential bar electrodes had a fixed 2 cm spacing and included a preamplifier with a gain of 10. sEMG signals were bandpass-filtered (20–250 Hz) and sampled at 2000 Hz using a 24-bit acquisition card (Noraxon Ultium, Noraxon, USA). Signals were rectified within a 200 ms moving window and normalized to maximal voluntary activation, accounting for intersubject differences in impedance [23]. In addition, the vastus medialis (VM) to vastus lateralis (VL) activation ratio was calculated to assess quadriceps balance relevant to patellar stability [49]. To assess the activation balance across the medial and lateral knee, we calculated two specific activation ratios: lateral hamstring to vastus lateralis (LH/VL) and medial hamstring to vastus medialis (MH/VM). Unlike conventional co-contraction indices that quantify absolute simultaneous agonist–antagonist activation as a sum, these ratios reflect the relative dominance of hamstrings over quadriceps on each aspect of the joint. As reported by Winby et al. and Sanchez-Ramirez et al. [50, 51], the LH/VL and MH/VM ratios are specifically associated with lateral and medial tibiofemoral compartment loading, respectively, whereas global co-contraction indices (e.g., overall hamstrings/quadriceps ratio) may obscure such aspect-specific differences. Given that KOA often involves compartments asymmetrically (e.g., predominant medial compartment involvement), separately assessing medial and lateral activation balance provides a more granular understanding of compartment-specific adaptations to disease progression. Accordingly, a higher LH/VL ratio indicates greater LH coactivation against the VL, suggesting a lateral stabilization strategy, while an elevated MH/VM ratio reflects MH dominance. Assessing these patterns helps clarify medial–lateral muscle balance and its association with knee torques, thereby revealing stress distribution across the medial and lateral compartments [52].

### Statistical analysis

Statistical analysis was performed using SPSS 26.0 (IBM Corp., Armonk, NY, USA). Data normality and variance were assessed using Kolmogorov–Smirnov and Levene’s tests. For intergroup comparisons between the affected limbs across the five groups (K-L I–IV and healthy controls), a one-way ANOVA was performed separately for males and females. When a significant main effect was detected, post hoc pairwise comparisons were conducted using Bonferroni correction to adjust for multiple comparisons (ten pairwise tests per ANOVA). To maintain table readability, only the overall ANOVA *p*-values (*p*1 for males, *p*2 for females) are shown in the tables, with the post hoc results denoted by superscript symbols (*). Comparisons between genders within each K-L grade employed an independent two-sample *t*-test, indicated in the tables by the symbol “α”. Correlations among gait, strength, and sEMG parameters, as well as with WOMAC scores, were evaluated using Spearman’s analysis. Multiple linear regression models with stepwise selection were constructed to simulate relationships between muscle coactivation, gait, strength, and to identify key factors associated with WOMAC subscales.

## Results

Table 1 summarizes the demographic characteristics of the subjects. Although height and weight differed between sexes, BMI showed no significant difference. The WOMAC scores of females are generally higher than those of males (*p* = 0.005–0.031).

### Gait analysis

Compared with the healthy control group, the KOA group exhibited significant reductions in speed, cadence, step length, stride length, and ROM at each joint (*p* < 0.001), along with a prolonged bilateral stance time (*p* =  < 0.001–0.003) (Table 2). Knee flexion moment (KFM) was significantly reduced in patients (*p* = 0.001–0.007), whereas knee adduction moment (KAM) and its impulse were significantly increased (*p* =  < 0.001–0.007). These alterations were more pronounced in females and in patients with K-L grade III or above (*p* < 0.001). Most parameters demonstrated specific trend changes with increasing K-L grade, and the trends observed in the contralateral side were consistent with, though less pronounced than, those in the affected side. As the K-L grade increased, the MoS, particularly the MoS-AP, tended to decrease (*p* < 0.001–0.006), whereas the ASI gradually increased, with ASI values consistently higher in males than in females (Table 2).

**Table 2:** Spatiotemporal, kinematic, and kinetic data of gait analysis in patients with KOA and healthy controls

Knee OA group (*n* = 165)									Control (*n* = 32)	*p*1	*p*2
	K-L I (*n* = 39)		K-L II (*n* = 38)		K-L III (*n* = 41)		K-L IV (*n* = 47)				
	Affected side	Contralateral side	Affected side	Contralateral side	Affected side	Contralateral side	Affected side	Contralateral side			
Speed (m/s) Male Female	1.20 ± 0.22^ɑ^ 0.97 ± 0.15*		1.03 ± 0.25^ɑ^* 0.82 ± 0.19*		0.88 ± 0.23^ɑ^* 0.69 ± 0.17*		0.68 ± 0.26^ɑ^* 0.49 ± 0.14*		1.29 ± 0.18 1.19 ± 0.11	< 0.001*	< 0.001*
Cadence (steps/min) Male Female	120.01 ± 5.31^ɑ^* 111.23 ± 5.56		112.34 ± 6.01^ɑ^* 104.72 ± 5.11*		103.51 ± 6.34^ɑ^* 93.08 ± 5.73*		91.27 ± 5.37^ɑ^* 82.76 ± 4.97*		124.22 ± 6.11^ɑ^ 116.17 ± 5.03	< 0.001*	< 0.001*
Step length (m) Male Female	0.61 ± 0.11 0.54 ± 0.12	0.63 ± 0.09 0.57 ± 0.07	0.57 ± 0.16^ɑ^ 0.47 ± 0.10*	0.60 ± 0.08 0.48 ± 0.07	0.47 ± 0.15* 0.39 ± 0.11*	0.54 ± 0.08 0.44 ± 0.05	0.40 ± 0.12^ɑ^* 0.33 ± 0.10*	0.47 ± 0.06 0.38 ± 0.05	0.65 ± 0.10^ɑ^ 0.57 ± 0.08	< 0.001*	< 0.001*
ASI_1_/ASI_2_	2.87 ± 2.24/2.73 ± 2.12		3.10 ± 2.39/2.83 ± 2.20		3.39 ± 2.52/3.08 ± 2.29		4.06 ± 2.80/3.80 ± 2.64				
Stance time (%) Male Female	60.11 ± 6.71 60.72 ± 6.17	58.07 ± 3.86 57.08 ± 4.06	62.51 ± 5.91 63.56 ± 5.73	64.62 ± 4.03 62.51 ± 4.09	65.65 ± 6.02* 65.41 ± 5.77	66.17 ± 4.22 66.38 ± 4.63	67.34 ± 5.73* 68.77 ± 3.93*	67.04 ± 4.12 69.89 ± 3.86	59.56 ± 5.72 62.38 ± 4.87	< 0.001*	0.003*
ASI_1_/ASI_2_	2.11 ± 1.58/1.72 ± 1.28		1.77 ± 1.32/1.72 ± 1.28		2.27 ± 1.67/1.90 ± 1.41		2.55 ± 1.79/2.28 ± 1.52				
ROM–knee (°) Male Female	60.33 ± 13.46 56.93 ± 14.68*	61.07 ± 11.29 56.07 ± 12.55	57.87 ± 15.66 53.74 ± 12.96*	60.56 ± 9.73 55.20 ± 10.75	51.60 ± 12.36* 47.39 ± 9.48*	57.77 ± 8.43 52.83 ± 9.90	45.71 ± 18.79* 40.08 ± 16.17*	51.77 ± 11.97 45.70 ± 13.24	62.51 ± 12.38 58.71 ± 10.27	< 0.001*	< 0.001*
ASI_1_/ASI_2_	3.24 ± 2.49/3.03 ± 2.32		3.27 ± 2.50/3.15 ± 2.40		3.69 ± 2.74/3.53 ± 2.60		4.34 ± 2.99/4.03 ± 2.81				
Peak KFM (Nm/(BW × HT)%) Male Female	3.49 ± 1.72 3.17 ± 1.61	3.55 ± 1.55 3.42 ± 1.53	3.21 ± 1.68 2.96 ± 1.50	3.37 ± 1.22 3.19 ± 1.24	2.73 ± 1.37 2.41 ± 1.26*	3.09 ± 1.09 2.98 ± 0.91	2.18 ± 1.22* 1.94 ± 1.04*	2.76 ± 0.98 2.35 ± 0.79	3.76 ± 1.57 3.41 ± 1.36	0.007*	0.001*
ASI_1_/ASI_2_	5.47 ± 4.32/5.36 ± 4.25		7.33 ± 5.04/7.16 ± 4.83		9.88 ± 5.47/9.45 ± 5.30		12.77 ± 5.79/13.84 ± 5.66				
Peak KAM (Nm/(BW × HT)%) Male Female	2.35 ± 0.76 2.73 ± 0.91	2.74 ± 0.62 2.76 ± 0.70	2.72 ± 0.93 2.96 ± 1.11	2.79 ± 0.52 3.23 ± 0.67	3.22 ± 1.38* 3.56 ± 1.47*	3.41 ± 1.41 3.78 ± 1.33	3.86 ± 1.66* 4.33 ± 1.96*	4.01 ± 1.39 4.41 ± 1.55	2.02 ± 0.75 2.61 ± 0.92	0.005*	< 0.001*
ASI_1_/ASI_2_	4.69 ± 3.58/4.36 ± 3.40		4.32 ± 3.30/4.13 ± 3.21		4.73 ± 3.59/4.54 ± 3.49		5.04 ± 3.83/4.97 ± 3.71				
MoS-AP (m) Male Female	0.32 ± 0.17 0.31 ± 0.15		0.27 ± 0.14* 0.25 ± 0.13		0.20 ± 0.11* 0.19 ± 0.08*		0.14 ± 0.08* 0.13 ± 0.08*		0.36 ± 0.15 0.34 ± 0.16	0.006*	0.004*
MoS-ML (m) Male Female	0.16 ± 0.09 0.15 ± 0.10		0.15 ± 0.09 0.13 ± 0.06		0.11 ± 0.07 0.09 ± 0.06*		0.07 ± 0.06* 0.06 ± 0.04*		0.17 ± 0.07 0.17 ± 0.05	< 0.001*	< 0.001*

Data are presented as mean ± standard deviation. K-L, Kellgren–Lawrence grade; RoM, range of motion in the sagittal plane; KFM, knee flexion moment; KAM, knee adduction moment; MoS, margins of stability; AP, anterior–posterior; ML, Medial–lateral; ASI, asymmetry index. ASI_1_ and ASI_2_ represent the limb symmetry of males and females, respectively. An ASI value of “0” indicates absolute symmetry between the limbs, whereas larger ASI values reflect greater asymmetry. Statistical value refers to ANOVA or the independent Student’s *t*-test. The overall ANOVA *p*-values (*p*1 for males, *p*2 for females) indicate whether significant differences exist among the five groups. *Indicates significant differences between KOA groups and the control group after Bonferroni correction. ^ɑ^Indicates significant difference between males and females within the same K-L grade (independent *t*-test, *p* < 0.05)

### Measures of muscle strength

Compared with the control group, the patients exhibited decreased values for APT, PTA, and AP during both knee flexion and extension (*p* < 0.001–0.043), whereas TPT and the H/Q ratio were increased (*p* < 0.001–0.046) (Table 3). These differences became more pronounced with higher K-L grades. Similar but less severe trends were observed in the contralateral limb. No sex-related differences were found in any of the measured parameters, except for APT in patients with K-L grade III and IV (*p* = 0.012–0.046). In addition, the LSI was higher in all KOA groups than in the control group, and males generally exhibited a higher LSI than females (Table 3).

**Table 3:** Data of an isokinetic muscle strength test in in patients with KOA and healthy controls (at 60°/s)

Knee OA group (*n* = 165)															Control (*n* = 32)	*p*1	*p*2
	K-L I (*n* = 39)		K-L II (*n* = 38)			K-L III (*n* = 41)					K-L IV (*n* = 47)						
	Affected side	Contralateral side	Affected side	Contralateral side		Affected side		Contralateral side		Affected side			Contralateral side				
Extension/quadriceps																	
APT (Nm/kg) Male Female	1.31 ± 0.41 1.17 ± 0.53	1.36 ± 0.33 1.20 ± 0.40	1.21 ± 0.48 1.04 ± 0.39	1.30 ± 0.23 1.11 ± 0.27		1.09 ± 0.50* 0.80 ± 0.39*		1.21 ± 0.30 1.04 ± 0.21			0.81 ± 0.38* 0.55 ± 0.27*		1.16 ± 0.29 0.96 ± 0.31		1.46 ± 0.31^ɑ^ 1.21 ± 0.31	0.005*	< 0.001*
LSI_1_/LSI_2_ (%)	92.4 ± 5.5/90.0 ± 5.4		82.9 ± 6.2/80.1 ± 6.4			72.1 ± 6.5/69.0 ± 6.9					60.2 ± 5.4/57.8 ± 5.8						
PTA (°) Male Female	58.73 ± 7.12 55.45 ± 8.37	59.56 ± 7.91 55.33 ± 8.25	52.90 ± 10.08* 50.45 ± 11.36	57.47 ± 9.52 54.17 ± 7.62		47.55 ± 14.86* 46.04 ± 13.26*		56.57 ± 10.48 53.29 ± 9.08			42.61 ± 17.35* 38.82 ± 15.56*		53.57 ± 11.58 51.36 ± 9.95		60.38 ± 12.72 56.74 ± 11.26	< 0.001*	< 0.001*
LSI_1_/LSI_2_ (%)	91.6 ± 5.5/89.4 ± 5.4		82.1 ± 6.2/79.4 ± 6.4			71.5 ± 6.4/68.6 ± 6.9					60.6 ± 5.5/58.4 ± 5.9						
Flexion/hamstring																	
APT (Nm/kg) Male Female	0.88 ± 0.30 0.79 ± 0.28	0.88 ± 0.25 0.80 ± 0.27	0.84 ± 0.35 0.71 ± 0.32		0.85 ± 0.26 0.75 ± 0.25		0.78 ± 0.35 0.56 ± 0.28		0.81 ± 0.28 0.72 ± 0.24			0.61 ± 0.31* 0.44 ± 0.23*		0.80 ± 0.30 0.68 ± 0.31	0.95 ± 0.32 0.80 ± 0.23	0.043*	< 0.001*
LSI_1_/LSI_2_ (%)	91.0 ± 5.5/88.4 ± 5.3		81.4 ± 6.1/78.4 ± 6.3				70.6 ± 6.5/67.4 ± 6.8					58.8 ± 5.3/56.3 ± 5.7					
PTA (°) Male Female	34.68 ± 7.82 35.26 ± 6.55	35.77 ± 5.56 34.43 ± 6.76	37.51 ± 8.46 39.41 ± 9.01		36.52 ± 6.11 35.47 ± 6.62		42.33 ± 12.38* 44.54 ± 15.88*		37.85 ± 9.03 35.82 ± 7.76			48.07 ± 18.81* 50.34 ± 23.29*		40.63 ± 5.21 39.70 ± 6.37	35.03 ± 5.32 33.74 ± 6.13	0.392	< 0.001*
LSI_1_/LSI_2_ (%)	91.3 ± 5.5/88.9 ± 5.3		81.9 ± 6.1/79.0 ± 6.3				71.2 ± 6.5/68.1 ± 6.8					59.4 ± 5.3/57.1 ± 5.7					
H/Q (%) Male Female	67.9 ± 13.3 68.2 ± 11.2	63.6 ± 10.1 65.3 ± 10.7	69.9 ± 12.3 67.3 ± 11.8		66.4 ± 13.4 66.5 ± 12.1		73.1 ± 10.8 73.5 ± 11.3		67.3 ± 12.7 69.7 ± 13.5			75.7 ± 10.4* 77.8 ± 7.5*		66.3 ± 10.5 69.8 ± 9.6	64.4 ± 9.2 65.5 ± 9.4	0.046*	< 0.001*
LSI_1_/LSI_2_ (%)	91.7 ± 5.5/89.2 ± 5.4		82.3 ± 6.2/79.4 ± 6.4				71.6 ± 6.5/68.5 ± 6.9					59.8 ± 5.4/57.5 ± 5.8					

Data are presented as mean ± standard deviation. K-L, Kellgren–Lawrence grade; APT, average peak torque; PTA, angle at peak torque; H/Q, the ratio of hamstring torque to quadriceps torque during concentric contraction; LSI, limb symmetry index. LSI_1_ and LSI_2_ represent the limb symmetry of males and females, respectively. Statistical value refers to ANOVA or the independent Student’s *t*-test. The overall ANOVA *p*-values (*p*1 for males, *p*2 for females) indicate whether significant differences exist among the five groups. *Indicates significant differences between KOA groups and the control group after Bonferroni correction ^ɑ^Indicates a significant difference between males and females within the same K-L grade (independent *t*-test, *p* < 0.05)

### sEMG combined with gait analysis

Significant coactivation was observed in both the antagonist and agonist muscles, predominantly during the early-, mid-, and late-stance phases, as well as the late swing phase (Table 4). No significant coactivation was detected in the other phases. Coactivation parameters exhibited inter-group differences (*p* =  < 0.001–0.048), whereas no significant gender differences were identified. The most pronounced coactivation of the hamstrings occurred during the early and late stance phases and the late swing phase (*p* < 0.001). In patients with KOA, the lateral coactivation ratio (LH/VL) was increased across all phases, with greater increases observed in patients with higher K-L grades (*p* = 0.002–0.048). In contrast, the medial coactivation ratio (MH/VM) showed only a slight increase during the late stance phase (*p* = 0.025). The VM/VL ratio was elevated during the early and late stance phases (*p* = 0.002) but decreased during the mid-stance and late swing phases (*p* < 0.001 and *p* = 0.023). The normalized activation levels of the anterior muscle group (VM + VL/VM + VL) and posterior muscle group (LH + MH/LH + MH) increased with higher K-L grades across all phases. Throughout all phases, LH activation was consistently greater than MH activation. No significant sex × group interaction was detected in the sEMG data, so a single *p*-value is presented in Table 4. The heightened lateral co-activation (LH/VL) across the gait cycle, together with the phase-dependent alteration in quadriceps balance (VM/VL), paralleled the reductions in speed and ROM, and coincided with the increased KAM observed in patients.

**Table 4:** sEMG data of patients with KOA and healthy controls synchronized with gait analysis

Knee OA group (*n* = 165)											Control (*n* = 32)	*p*
	K-L I (*n* = 39)		K-L II (*n* = 38)			K-L III (*n* = 41)			K-L IV (*n* = 47)			
	Affected side	Contralateral side	Affected side	Contralateral side	Affected side		Contralateral side	Affected side		Contralateral side		
Early stance phase												
LH/VL	0.60 ± 0.18	0.60 ± 0.11	0.68 ± 0.24	0.62 ± 0.16	0.76 ± 0.29*		0.65 ± 0.15	0.81 ± 0.38*		0.69 ± 0.18	0.57 ± 0.12	0.010*
MH/VM	0.64 ± 0.23	0.65 ± 0.16	0.69 ± 0.27	0.64 ± 0.21	0.70 ± 0.33		0.68 ± 0.18	0.77 ± 0.36		0.70 ± 0.20	0.68 ± 0.19	0.301
LH/MH	1.29 ± 0.48	1.22 ± 0.40	1.43 ± 0.56	1.35 ± 0.51	1.56 ± 0.64*		1.41 ± 0.49	1.83 ± 0.83*		1.60 ± 0.31	1.17 ± 0.45	0.001*
VM/VL	0.72 ± 0.15	0.75 ± 0.18	0.76 ± 0.21	0.77 ± 0.22	0.82 ± 0.29		0.61 ± 0.25	0.91 ± 0.35*		0.64 ± 0.22	0.74 ± 0.16	0.002*
LH + MH/*LH* + *MH*	0.23 ± 0.13	0.20 ± 0.08	0.27 ± 0.15	0.21 ± 0.06	0.32 ± 0.21		0.24 ± 0.11	0.40 ± 0.29*		0.27 ± 0.16	0.21 ± 0.10	< 0.001*
VM + VL/*VM* + *VL*	0.37 ± 0.15	0.35 ± 0.13	0.40 ± 0.19	0.36 ± 0.12	0.44 ± 0.25		0.35 ± 0.16	0.51 ± 0.24*		0.37 ± 0.11	0.36 ± 0.11	0.012*
Mid-stance phase												
LH/VL	0.72 ± 0.21	0.70 ± 0.16	0.74 ± 0.26	0.71 ± 0.19	0.79 ± 0.30		0.73 ± 0.15	0.85 ± 0.37*		0.77 ± 0.13	0.71 ± 0.19	0.048*
MH/VM	0.73 ± 0.23	0.73 ± 0.19	0.77 ± 0.28	0.74 ± 0.21	0.83 ± 0.33		0.77 ± 0.25	0.86 ± 0.42		0.82 ± 0.31	0.76 ± 0.29	0.526
LH/MH	1.30 ± 0.63	1.26 ± 0.62	1.37 ± 0.68	1.28 ± 0.56	1.55 ± 0.79		1.30 ± 0.25	1.70 ± 0.82*		1.30 ± 0.29	1.31 ± 0.62	0.010*
VM/VL	0.71 ± 0.14	0.72 ± 0.15	0.68 ± 0.17	0.73 ± 0.12	0.62 ± 0.13		0.74 ± 0.14	0.56 ± 0.30*		0.70 ± 0.16	0.72 ± 0.20	< 0.001*
LH + MH/*LH* + *MH*	0.13 ± 0.06	0.10 ± 0.05	0.15 ± 0.07	0.12 ± 0.06	0.18 ± 0.10*		0.13 ± 0.05	0.23 ± 0.17*		0.16 ± 0.07	0.11 ± 0.04	0.021*
VM + VL/*VM* + *VL*	0.17 ± 0.09	0.15 ± 0.08	0.20 ± 0.11	0.17 ± 0.07	0.23 ± 0.12*		0.18 ± 0.10	0.26 ± 0.19*		0.21 ± 0.10	0.16 ± 0.06	< 0.001*
Late stance phase												
LH/VL	0.81 ± 0.36	0.86 ± 0.30	0.91 ± 0.41	0.88 ± 0.36	1.17 ± 0.53*		0.96 ± 0.29	1.28 ± 0.66*		1.02 ± 0.50	0.82 ± 0.31	0.027*
MH/VM	0.53 ± 0.27	0.55 ± 0.25	0.60 ± 0.30	0.57 ± 0.27	0.70 ± 0.34		0.61 ± 0.26	0.76 ± 0.39*		0.60 ± 0.28	0.53 ± 0.24	0.025*
LH/MH	2.01 ± 0.96	2.14 ± 0.88	2.26 ± 1.17	2.10 ± 0.91	2.44 ± 1.36		2.22 ± 1.05	2.65 ± 1.35*		2.25 ± 1.24	2.11 ± 0.75	0.021*
VM/VL	0.70 ± 0.25	0.71 ± 0.30	0.73 ± 0.32	0.70 ± 0.25	0.76 ± 0.35		0.75 ± 0.28	0.81 ± 0.42*		0.76 ± 0.34	0.72 ± 0.27	0.002*
LH + MH/*LH* + *MH*	0.07 ± 0.03	0.09 ± 0.05	0.12 ± 0.07	0.10 ± 0.04	0.19 ± 0.11*		0.15 ± 0.09	0.31 ± 0.19*		0.18 ± 0.10	0.05 ± 0.03	< 0.001*
VM + VL/*VM* + *VL*	0.10 ± 0.07	0.11 ± 0.05	0.13 ± 0.08	0.10 ± 0.05	0.18 ± 0.10*		0.13 ± 0.09	0.26 ± 0.17*		0.18 ± 0.12	0.08 ± 0.05	< 0.001*
Late swing phase												
LH/VL	0.24 ± 0.13	0.20 ± 0.15	0.25 ± 0.15	0.22 ± 0.08	0.30 ± 0.17*		0.25 ± 0.11	0.38 ± 0.19*		0.28 ± 0.16	0.22 ± 0.07	0.002*
MH/VM	0.20 ± 0.09	0.16 ± 0.05	0.21 ± 0.11	0.18 ± 0.10	0.23 ± 0.14		0.20 ± 0.11	0.25 ± 0.18		0.17 ± 0.09	0.17 ± 0.14	0.521
LH/MH	1.63 ± 0.76	1.58 ± 0.73	1.70 ± 0.95	1.64 ± 0.86	1.88 ± 1.04		1.71 ± 0.85	2.20 ± 1.12*		1.80 ± 0.97	1.64 ± 0.70	0.009*
VM/VL	0.75 ± 0.30	0.72 ± 0.25	0.71 ± 0.36	0.70 ± 0.28	0.65 ± 0.31		0.68 ± 0.33	0.59 ± 0.30*		0.73 ± 0.35	0.78 ± 0.26	0.023*
LH + MH/*LH* + *MH*	0.11 ± 0.07	0.09 ± 0.06	0.13 ± 0.09	0.12 ± 0.07	0.19 ± 0.12*		0.17 ± 0.10	0.25 ± 0.17*		0.18 ± 0.13	0.10 ± 0.06	< 0.001*
VM + VL/*VM* + *VL*	0.25 ± 0.15	0.24 ± 0.17	0.30 ± 0.19	0.25 ± 0.20	0.36 ± 0.20*		0.27 ± 0.17	0.40 ± 0.22*		0.31 ± 0.16	0.22 ± 0.10	< 0.001*

Data are presented as mean ± standard deviation. K-L, Kellgren–Lawrence grade; LH, lateral hamstring; VL,vastus lateralis; VM, vastus medialis; MH, medial hamstring; *LH* + *MH*, the sum of the maximum surface electromyography amplitudes of the medial and lateral hamstrings during knee flexion in isokinetic muscle strength testing; *VM* + *VL*, the sum of the maximum surface electromyography amplitudes of the vastus medialis and vastus lateralis during knee extension in isokinetic muscle strength testing. The overall ANOVA *p*-values indicate whether significant differences exist among the five groups, with sex included as a covariate where appropriate (no significant sex × group interaction was detected). The resulting *p*-values are presented as a single column. *Indicates significant differences between KOA groups and the control group after Bonferroni correction

### sEMG combined with strength test

During knee extension, the VM activation was higher than VL activation. However, the VM/VL ratio was significantly reduced in patients with KOA, and this reduction became more pronounced with increasing K-L grade (*p* = 0.007) (Table 5). The ratios of posterior to anterior muscle activation (MH + LH/VM + VL), as well as the normalized activation ratios of the MH and LH (MH/*MH* and LH/*LH*), were increased in the patient group, and these ratios increased with higher K-L grades (*p* = 0.002, 0.012, and 0.022). During knee flexion, the medial muscles activation (VM and MH) were lower than the lateral muscles activation (VL and LH) in all subjects. The VM/VL and MH/LH ratios were significantly lower in patients with KOA, with greater reductions observed at higher K-L grades (*p* = 0.017 and 0.026). Conversely, the ratio of anterior to posterior muscle activation (VM + VL/MH + LH) and the normalized VL activation (VL/*VL*) were significantly higher in the patient group, and both increased with advancing K-L grade (*p* = 0.032 and 0.018). No significant sex × group interaction was detected in the sEMG data, so a single *p*-value is presented in Table 5. The shift toward greater lateral and posterior muscle activation during both extension and flexion corresponds to the lower APT and higher H/Q ratio in the KOA group, suggesting a functional imbalance that may compromise net force output during strength tasks.

**Table 5:** sEMG data of patients with KOA and healthy controls synchronized with an isokinetic muscle strength test

Knee OA group (*n* = 165)										Control (*n* = 32)	*p*
	K-L I (*n* = 39)			K-L II (*n* = 38)		K-L III (*n* = 41)		K-L IV (*n* = 47)			
	Affected side	Contralateral side		Affected side	Contralateral side	Affected side	Contralateral side	Affected side	Contralateral side		
Extension at 60°/s											
VM/VL	1.31 ± 0.70		1.30 ± 0.65	1.21 ± 0.66	1.26 ± 0.56	1.01 ± 0.52	1.19 ± 0.58	0.83 ± 0.47*	1.08 ± 0.56	1.36 ± 0.66	0.007*
MH/LH	0.66 ± 0.29		0.54 ± 0.20	0.65 ± 0.33	0.51 ± 0.22	0.61 ± 0.35	0.52 ± 0.22	0.62 ± 0.37	0.47 ± 0.20	0.67 ± 0.23	0.320
MH + LH/VM + VL	0.11 ± 0.07		0.09 ± 0.05	0.13 ± 0.07	0.11 ± 0.06	0.17 ± 0.10*	0.11 ± 0.07	0.21 ± 0.12*	0.13 ± 0.09	0.10 ± 0.06	0.002*
MH/*MH*	0.18 ± 0.08		0.15 ± 0.07	0.20 ± 0.13	0.18 ± 0.10	0.23 ± 0.13*	0.18 ± 0.08	0.24 ± 0.15*	0.20 ± 0.11	0.17 ± 0.10	0.012*
LH/*LH*	0.15 ± 0.09		0.11 ± 0.06	0.18 ± 0.10	0.13 ± 0.06	0.22 ± 0.14*	0.15 ± 0.10	0.27 ± 0.19*	0.15 ± 0.12	0.13 ± 0.08	0.022*
Flexion at 60°/s											
VM/VL	0.59 ± 0.24		0.62 ± 0.28	0.51 ± 0.25	0.63 ± 0.30	0.45 ± 0.24	0.57 ± 0.26	0.36 ± 0.21*	0.55 ± 0.29	0.63 ± 0.27	0.017*
MH/LH	0.72 ± 0.45		0.78 ± 0.41	0.65 ± 0.35	0.75 ± 0.42	0.59 ± 0.30	0.68 ± 0.35	0.49 ± 0.35*	0.69 ± 0.37	0.76 ± 0.40	0.026*
VM + VL/MH + LH	0.32 ± 0.20		0.27 ± 0.15	0.34 ± 0.22	0.30 ± 0.16	0.36 ± 0.20	0.29 ± 0.19	0.38 ± 0.22*	0.33 ± 0.15	0.28 ± 0.13	0.032*
VM/*VM*	0.09 ± 0.07		0.10 ± 0.06	0.11 ± 0.08	0.07 ± 0.07	0.10 ± 0.07	0.05 ± 0.06	0.10 ± 0.11	0.09 ± 0.07	0.08 ± 0.06	0.286
VL/*VL*	0.20 ± 0.13		0.15 ± 0.07	0.23 ± 0.15	0.20 ± 0.14	0.25 ± 0.15	0.21 ± 0.10	0.28 ± 0.17*	0.20 ± 0.13	0.18 ± 0.10	0.018*

Data are presented as mean ± standard deviation. K-L, Kellgren–Lawrence grade; LH, lateral hamstring; VL, vastus lateralis; VM, vastus medialis; MH, medial hamstring; *MH* and *LH*, the maximum surface electromyography amplitudes of the medial and lateral hamstrings during knee flexion in isokinetic muscle strength testing; *VM* and *VL*, the maximum surface electromyography amplitudes of the vastus medialis and vastus lateralis during knee extension in isokinetic muscle strength testing. The overall ANOVA *p*-values indicate whether significant differences exist among the five groups, with sex included as a covariate where appropriate (no significant sex × group interaction was detected). The resulting *p*-values are presented as a single column. *Indicates a significant difference between KOA groups and the control group after Bonferroni correction

### Correlation analysis

Pain scores were significantly positively correlated with peak KAM, KAM impulse, and the LH/VL ratio during early stance (*r* = 0.532–0.584, *p* < 0.05), and moderately correlated with the remaining 16 parameters (|*r*|= 0.365–0.463, *p* < 0.05) (Table S1). Stiffness scores were significantly negatively correlated with ROM-knee and peak KFM (*r* = −0.542 and −0.565, *p* < 0.05), and moderately correlated with nine other parameters (|*r*|= 0.361–0.488, *p* < 0.05). Functional scores were significantly positively correlated with speed and ROM-knee (*r* = 0.580 and 0.557, *p* < 0.05), and moderately correlated with seven other parameters (|*r*|= 0.368–0.451, *p* < 0.05). Furthermore, the LH/VL ratio during early stance was positively correlated with peak KAM and KAM impulse (*r* = 0.598 and 0.615, *p* < 0.05) (Table S3). The VM + VL/*VM* + *VL* ratio was significantly negatively correlated with step length in the late swing phase (*r* = −0.551, *p* < 0.05). Approximately 20 parameter pairs exhibited moderate correlations between gait parameters and sEMG parameters (|*r*|= 0.365–0.492, *p* < 0.05) (Table S3). Only the MH + LH/VM + VLe ratio vastus lateralis activation (VLe) was significantly negatively correlated with average peak torque (APTe) (*r* = −0.575, *p* < 0.05), and an additional ten parameter pairs demonstrated moderate associations between muscle strength parameters and sEMG parameters (|*r*|= 0.360–0.484, *p* < 0.05) (Table S5).

### Multivariate linear regression

On the basis of parameters exhibiting significance in Spearman correlation analyses, we constructed multiple linear regression models for WOMAC pain, stiffness, and function scores. Given the significant impact of BMI on the KOA severity, although BMI did not correlate with the WOMAC scores, it was still incorporated into the models for further investigation. In the WOMAC-pain model, a total of ten parameters, including speed, peak KAM, and ROM-knee, reached statistical significance (*p* =  < 0.001–0.043). For the WOMAC-stiffness and WOMAC-function models, six and eight parameters were statistically significant, respectively (*p* =  < 0.001–0.031 and *p* = 0.003–0.046) (Table S2). Separate regression analyses were also performed for sEMG, gait, and muscle strength parameters. Table S4 summarizes 15 models derived from gait and muscle strength tests, wherein each model contained 1–4 sEMG parameters that were statistically significant (*p* = 0.004–0.047).

## Discussion

This study demonstrates that unilateral KOA patients exhibit significant bilateral alterations in muscle activation, gait patterns, and muscle strength. Most of the biomechanical parameters show marked asymmetry between the limbs. These characteristics become more pronounced with increasing disease severity and correlate with patient-reported outcomes. Our multimodal assessment identifies significant correlations among the measured parameters, providing new insight into the relationship between neuromuscular function and disease progression in KOA.

Consistent with previous studies [22, 52–56], pain, stiffness, and functional limitation progressed with increasing K-L grade. Females in this cohort reported more severe symptoms. As disease severity advanced, most gait and strength parameters deteriorated progressively, with more pronounced abnormalities observed in females. Specifically, in patients with KOA, the the KAM and KAM impulse increased by approximately 11.6–78.1% and 10.0–75.0%, while KFM decreased by approximately 7.8–42.7%, respectively. Based on the results of Erhart-Hledik et al., compared with patients with mild KOA, the severe KOA group exhibited a 21.8% increase in peak KAM and a 6.4% reduction in KFM [60]. This pattern appears to be a characteristic gait manifestation of KOA [57–60]. We also found that peak KAM increased with higher K-L grades and was significantly positively correlated with pain scores, consistent with findings reported by Hall et al. This suggest that peak KAM may be associated with biomechanical alterations during disease progression [58]. This change was also more evident in females, which may partially explain their greater symptom severity. Regression analysis further revealed that elevated peak KAM, pain, and enhanced hamstring coactivation were interrelated, particularly regarding increased activation of LH. Studies by Heiden et al. and Lewek et al. indicated that enhanced LH activation was sensitive to K-L grade and significantly correlated with higher peak KAM [59, 61]. On the basis of these findings [53, 59, 61, 62], we hypothesized that excessive LH activation may be associated with increased tibial external rotation and medial compartment loading, with peak KAM serving as a sensitive indicator of medial compartment loading [19]. This seems to reveal a vicious cycle regarding increased coactivation, elevated medial compartment stress, pain, and KOA severity. Furthermore, the reduction in KFM was associated with increased coactivation of the anterior and posterior muscle groups. This is likely because simultaneous contraction of these muscle groups reduces the net flexion force, while KFM represents the net torque required to counteract gravity during early stance [59, 60].

sEMG of gait synchronization revealed increased coactivation of the quadriceps and hamstrings across all four gait phases in patients. This compensatory activation was particularly pronounced during early stance and late swing phases, especially in the lateral muscle group (LH and VL). The mean ratio of LH/MH PP1 reported by Rutherford et al. was 75.6% higher in patients compared with healthy controls [63], whereas the corresponding value in our study ranged from approximately 14.6% to 33.3%. Although the intensity and timing of observed antagonistic coactivation varied slightly across investigations [13, 52, 53, 62, 63], the prevailing consensus is that, in the early stages of KOA, this represents a compensatory strategy that sacrifices muscle activation efficiency to achieve greater joint stability [64–67]. Specifically, increased coactivation during the stance subphases was associated with gait parameters such as reduced speed, decreased cadence, and prolonged stance duration, and was also significantly correlated with symptoms of pain and stiffness [68]. In this regard, we concur with Hatfield et al., while joint stability is enhanced, this manifests as a typical “stiff knee” gait pattern, which may delay stress unloading and be associated with diminished net propulsive force during the propulsion phase [13, 69]. During late-swing phase, excessive coactivation was associated with shorter step length, reduced ROM-knee, and a narrower MoS-AP. This pre-heel-strike stabilization strategy may increase joint stiffness to counteract gravitational loads, but it restricts limb advancement and joint mobility [19, 59–63, 69]. Based on our observations, many patients with severe KOA often lean the trunk forward in late swing to shift the gravity center anteriorly. While this may partially compensate for insufficient step length and range of motion, it can compromise postural stability and reduce the MoS [52, 59, 60, 70–75]. A reduced MoS in patients may suggest a substantially increased risk of falls during walking [38–41].

The H/Q ratio is an essential indicator for assessing the balance of periarticular musculature. Our findings revealed that the H/Q ratio increased by 4.8–15.5% in patients with KOA. In comparison, Patsika et al. reported a 34.4% increase [77]. An elevated H/Q ratio in patients suggests an imbalance between hamstrings and quadriceps strength during joint movement [76]. This finding was further corroborated by sEMG, which demonstrated that activation of the MH, LH, and combined hamstrings relative to the quadriceps increased by 10.0–110.0% during knee extension in patients with different K-L grades. Although Segal et al. did not include healthy controls, their self-controlled analysis in patients with KOA indicated that increased hamstring coactivation reduced quadriceps strength and accelerated cartilage degeneration in a sex-specific manner [5, 16]. The present study also identified a comparable increase in coactivation of the VL and VM as antagonists during knee flexion. To our knowledge, this specific finding has not been previously reported. And the underlying purposes of these two coactivation patterns may differ. Enhanced hamstring activation during knee extension may increase the capacity of the muscles to oppose anterior tibial translation, thereby potentially enhancing sagittal plane joint stability when required. Conversely, the increased quadriceps activation during knee flexion may be related to a strategy to avoid flexion-induced pain. Both scenarios may reflect a strategy wherein activation efficiency is compromised to gain stability and symptom relief [5, 16, 53, 78], potentially analogous to the gait strategies we analyzed previously. Furthermore, we observed a potential alteration in the balance of quadriceps contraction. In healthy subjects, knee extension is characterized by dominant activation of the VM over the VL. In patients with KOA, this pattern may be reversed, with VL activation becoming predominant. This specific pattern persisted during knee flexion. Our results differ from those reported by Patsika et al. [77], who observed increased VM activation in female patients, which they attributed to a mechanism for stabilizing the patella and reducing medial compartment loading. We hypothesize that the enhanced VL activation observed across all K-L grades may be related to patellar stabilization and patellofemoral pressure balance [52, 77, 79]. We also found an association between this imbalance and pain during early stance phase of gait. However, as reported by Wu et al. [79], patellofemoral osteoarthritis and alignment may be important factors to quadriceps contraction imbalance. Therefore, it cannot be definitively determined whether this change is solely attributable to KOA. These discrepancies in findings suggest that, in addition to the factors considered by Patsika et al., the status of patellofemoral joint pathology is crucial for analyzing quadriceps activation patterns. Moreover, regression analyses indicated that strength parameters, including APT, PTA, TPT, and AP, were associated with increased coactivation. These findings are partially consistent with previous studies [2, 4–6, 10, 16, 18]. We speculate that excessive antagonistic coactivation partially counteracts the torque generated by the agonist muscles during flexion and extension, thereby reducing the resultant net muscle output.

This study also yielded several secondary findings. Patient-reported outcomes, in addition to being associated with the aforementioned activation patterns, were correlated with speed, APT, and ROM-knee, which is consistent with previous literature [54, 80]. Furthermore, sEMG parameters did not exhibit significant sex differences. Aligned with prior studies, we propose that although muscle activation may be similar between sexes, the resulting mechanical manifestations (e.g., muscle strength and gait) display sex differences, which are likely attributable to morphological and physiological disparities in the musculoskeletal system (the “executor”) rather than differences in neural drive (the “commander”) [6, 81–85]. Interestingly, among all 56 indicators, only 4 showed significant differences in K-L II patients compared with the control group, whereas 26 indicators were significantly different in patients with K-L grade III. We hypothesize that the transition from grade II to grade III may represent an accelerated phase of functional deterioration. This underscores the importance of initiating early, targeted prehabilitation prior to this stage, potentially improving clinical outcomes in KOA management [86, 87].

This study has several limitations. Firstly, its cross-sectional design establishes associations, not absolute causality, between neuromuscular parameters and KOA severity. By including the full K-L grade spectrum, we provide indirect evidence of progression, but longitudinal studies are still needed to confirm causal pathways. Moreover, laboratory-based testing may reduce ecological validity despite ensuring high data precision and comparability. Future work should incorporate wearable sensors in free-living environments. Finally, sEMG reflects neural drive, not direct mechanical output, as force generation is modulated by factors such as muscle–tendon stiffness and fatigue. The observed discordance between sEMG and strength metrics, captured via synchronous multimodal assessment, itself highlights the role of peripheral muscle–tendon properties in KOA pathology.

## Conclusions

KOA is not only characterized by gait abnormalities and decreased muscle strength but may also be associated with an adaptive neuromuscular strategy. This strategy is primarily defined by excessive coactivation of the hamstrings, particularly the lateral hamstrings, with the vastus lateralis. Although this coactivation may initially serve as a compensatory response to joint instability and structural degeneration, it is associated with gait abnormalities, muscle strength decline, and worsening of symptoms. Muscle coactivation may serve as a promising biomechanical marker to enhance our understanding of the clinical manifestations of KOA.

## Supplementary Information

Additional file 1.

## Data Availability

The data that support the findings of this study are not openly available due to reasons of sensitivity and are available from the corresponding author upon reasonable request. The data are located in a controlled-access data storage at the Army Medical University of the People’s Liberation Army of China. The original data of this study can be provided upon reasonable request, and such requests must be approved by the relevant institutions.
