# Supplementary material for: Associations of muscle coactivation patterns with gait, muscle strength, and symptoms across different stages of knee osteoarthritis
Source: Knee Surg Relat Res. 2026 Jul 6;38:27. doi: 10.1186/s43019-026-00326-4 (PMC13339328; doi:10.1186/s43019-026-00326-4)
Supplement: Supplementary file 1 — Additional file 1. [file 43019_2026_326_MOESM1_ESM.docx]

**The main inclusion and exclusion criteria：**

The main inclusion criteria for participation included: (1) a clinical diagnosis of KOA based on the criteria provided by the American College of Rheumatology [29], confirmed by knee X-rays and physical examination, (2) age between 50 and 75 years, and (3) radiological assessment of both knees in the standing anteroposterior and lateral positions, and classification using the Kellgren-Lawrence (K-L) criteria scale, with ≥1 grade considered as KOA [30]. All individuals meeting K-L grades I-IV were recruited. Exclusion criteria included: (1) having undergone any lower extremity surgery, (2) any additional orthopedic problems diagnosed in the hip, knee, or ankle; (3) neurological disorders (e.g., Parkinson's disease, vertigo, or stroke), or (4) individuals unable to stand or walk, or those who had received other forms of joint treatment within the past two months.

Table S1. Correlation analysis of parameters and WOMAC scores on the affected side

| Parameters | WOMAC Pain  r | WOMAC Stiffness  r | WOMAC Function  r |
| --- | --- | --- | --- |
|  |  |  |  |
| BMI | 0.214 | 0.103 | 0.069 |
| Gait analysis | | | |
| Speed | -0.463 | -0.159 | -0.580 |
| Cadence | -0.038 | -0.024 | -0.109 |
| Step length | -0.183 | -0.262 | -0.378 |
| Stance time | 0.380 | 0.148 | 0.075 |
| ROM-hip | -0.306 | -0.263 | -0.198 |
| ROM-knee | -0.391 | -0.542 | -0.557 |
| ROM-ankle | -0.030 | -0.058 | -0.288 |
| Peak KFM | -0.477 | -0.565 | -0.448 |
| Peak KAM | 0.532 | 0.304 | 0.184 |
| KAM impulse | 0.584 | 0.294 | 0.263 |
| MoS-AP | -0.084 | -0.367 | -0.051 |
| MoS-ML | -0.019 | -0.056 | -0.087 |
| Isokinetic muscle strength test | | | |
| APTe | -0.040 | -0.122 | -0.368 |
| PTAe | -0.267 | -0.380 | -0.089 |
| TPTe | 0.031 | 0.108 | 0.144 |
| APe | -0.189 | -0.024 | -0.038 |
| APTf | -0.218 | -0.328 | -0.375 |
| PTAf | 0.298 | 0.393 | 0.305 |
| TPTf | 0.194 | 0.257 | 0.278 |
| APf | -0.153 | -0.157 | -0.273 |
| H/Q | 0.370 | 0.361 | 0.451 |
| sEMG combined with gait analysis | | | |
| Early stance phase | | | |
| LH/VL | 0.576 | 0.305 | 0.127 |
| MH/VM | 0.107 | 0.129 | 0.226 |
| LH/MH | 0.307 | 0.221 | 0.232 |
| VM/VL | 0.443 | 0.117 | 0.270 |
| LH+MH/*LH+MH* | 0.433 | 0.488 | 0.275 |
| VM+VL/*VM+VL* | 0.081 | 0.212 | 0.128 |
| Mid-stance phase | | | |
| LH/VL | 0.291 | 0.108 | 0.270 |
| MH/VM | 0.022 | 0.176 | 0.217 |
| LH/MH | 0.216 | 0.135 | 0.205 |
| VM/VL | 0.109 | 0.185 | 0.194 |
| LH+MH/*LH+MH* | 0.406 | 0.410 | 0.248 |
| VM+VL/*VM+VL* | 0.422 | 0.446 | 0.296 |
| Late stance phase | | | |
| LH/VL | 0.408 | 0.272 | 0.244 |
| MH/VM | 0.063 | 0.189 | 0.014 |
| LH/MH | 0.299 | 0.145 | 0.071 |
| VM/VL | 0.088 | 0.159 | 0.011 |
| LH+MH/*LH+MH* | 0.461 | 0.238 | 0.401 |
| VM+VL/*VM+VL* | 0.159 | 0.369 | 0.064 |
| Late swing phase |  |  |  |
| LH/VL | 0.365 | 0.177 | 0.244 |
| MH/VM | 0.141 | 0.033 | 0.159 |
| LH/MH | 0.242 | 0.229 | 0.233 |
| VM/VL | 0.130 | 0.201 | 0.113 |
| LH+MH/*LH+MH* | 0.449 | 0.388 | 0.266 |
| VM+VL/*VM+VL* | 0.074 | 0.187 | 0.428 |
| sEMG combined with isokinetic muscle strength test | | | |
| VM/VLe | -0.286 | -0.088 | -0.108 |
| MH/LHe | -0.116 | -0.150 | -0.020 |
| MH+LH/VM+VLe | 0.414 | 0.195 | 0.023 |
| MH/*MH*e | 0.105 | 0.219 | 0.059 |
| LH/*LH*e | 0.399 | 0.256 | 0.262 |
| VM/VLf | -0.024 | -0.148 | -0.142 |
| MH/LHf | -0.046 | -0.209 | -0.140 |
| VM+VL/MH+LHf | 0.117 | 0.243 | 0.121 |
| VM/*VM*f | 0.227 | 0.271 | 0.170 |
| VL/*VL*f | 0.368 | 0.047 | 0.008 |

e and f respectively represent the extension and flexion phases of the knee during the isokinetic muscle strength test. *Indicates a significant correlation at P < 0.05

Table S2. Multivariate regression analysis between various examination parameters and WOMAC scores

| Parameters | *B* | *β* | *P* value | 95%CI |
| --- | --- | --- | --- | --- |
| WOMAC-pain regression model | | | |  |
| Speed | -3.084 | -0.157 | 0.002 | -4.617，-1.551 |
| Peak KAM | 3.385 | 0.982 | ＜0.001 | 1.661，5.109 |
| LH/VL - Early stance | 7.350 | 0.418 | 0.005 | 3.588，11.112 |
| LH+MH/*LH+MH -* Early stance | 5.217 | 0.218 | 0.017 | 1.244，9.190 |
| VM/VL - Early stance | 5.010 | 0.266 | 0.026 | 0.999，9.021 |
| VM+VL/*VM+VL* -Mid-stance | 3.725 | 0.104 | 0.038 | 1.080，6.370 |
| LH+MH/*LH+MH* -late stance | 4.783 | 0.140 | 0.011 | 1.407，8.159 |
| LH/*LH*e | 5.833 | 0.163 | 0.043 | 0.698，10.968 |
| WOMAC-stifness regression model | | | | |
| ROM-knee | -0.085 | -0.468 | ＜0.001 | -0.129，-0.041 |
| Peak KFM | -0.135 | -0.068 | ＜0.001 | -0.197，-0.073 |
| LH+MH/*LH+MH -* Early stance | 5.270 | 0.391 | 0.009 | 1.894，8.646 |
| VM+VL/*VM+VL -* Mid-stance | 6.778 | 0.338 | 0.016 | 2.126，11.430 |
| VM+VL*/VM+VL -* Late stance | 8.534 | 0.379 | 0.031 | 1.800，15.268 |
| LH+MH*/LH+MH -* Late swing | 4.686 | 0.213 | 0.005 | 1.728，7.644 |
| WOMAC-function regression model | | | | |
| Speed | -5.388 | -0.105 | 0.021 | -8.718，-2.058 |
| ROM-knee | -0.629 | -0.743 | 0.003 | -1.039，-0.219 |
| Step length | -4.667 | -0.049 | 0.021 | -7.634，-1.700 |
| Peak KFM | -8.314 | -0.899 | 0.032 | -14.162，-2.466 |
| APTe | -6.572 | -0.238 | 0.046 | -12.698，-0.446 |
| H/Q | 1.033 | 0.857 | 0.013 | 0.330，1.736 |
| LH+MH/*LH+MH* - Late stance | 6.480 | 0.072 | 0.025 | 2.608，10.352 |
| VM+VL/*VM+VL* - Late swing | 5.416 | 0.079 | 0.043 | 0.608，10.224 |

e and f respectively represent the extension and flexion phases of the knee during the isokinetic muscle strength test. CI, confidence interval.

Table S3. Correlation analysis of gait parameters and sEMG parameters on the affected side

| Parameters | Speed | Cadence | Step length | Stance time | ROM-hip | ROM-knee | ROM-ankle | Peak KFM | Peak KAM | KAM impulse | MoS-AP | MoS-ML |
| --- | --- | --- | --- | --- | --- | --- | --- | --- | --- | --- | --- | --- |
| Early stance phase | | | | | | | | | | | | |
| LH/VL | -0.246 | -0.088 | -0.112 | 0.154 | -0.194 | -0.211 | -0.057 | -0.304 | 0.598 | 0.615 | -0.313 | -0.315 |
| MH/VM | -0.082 | -0.220 | -0.164 | 0.074 | -0.254 | -0.250 | -0.007 | -0.210 | 0.017 | 0.219 | -0.170 | -0.154 |
| LH/MH | -0.082 | -0.226 | -0.314 | 0.144 | -0.120 | -0.173 | -0.060 | -0.296 | 0.405 | 0.385 | -0.189 | -0.170 |
| VM/VL | -0.017 | -0.107 | -0.056 | 0.324 | -0.044 | -0.26 | -0.137 | -0.126 | 0.165 | 0.014 | -0.249 | -0.148 |
| LH+MH/LH+MH | -0.24 | -0.062 | -0.123 | 0.078 | -0.230 | -0.119 | -0.099 | -0.454 | 0.492 | 0.470 | -0.102 | -0.140 |
| VM+VL/VM+VL | -0.109 | -0.247 | -0.217 | 0.097 | -0.070 | -0.194 | -0.112 | -0.455 | 0.258 | 0.075 | -0.180 | -0.222 |
| Mid-stance phase | | | | | | | | | | | | |
| LH/VL | -0.272 | -0.223 | -0.253 | 0.279 | -0.103 | -0.041 | -0.208 | -0.284 | 0.124 | 0.319 | -0.244 | -0.262 |
| MH/VM | -0.188 | -0.112 | -0.004 | 0.194 | -0.062 | -0.026 | -0.225 | -0.192 | 0.087 | 0.084 | -0.168 | -0.184 |
| LH/MH | -0.329 | - 0.285 | -0.073 | 0.335 | -0.163 | -0.025 | -0.094 | -0.203 | 0.219 | 0.263 | -0.229 | -0.198 |
| VM/VL | 0.164 | 0.241 | 0.133 | -0.127 | 0.224 | 0.221 | 0.037 | 0.202 | -0.011 | -0.245 | 0.099 | 0.066 |
| LH+MH/*LH+MH* | -0.282 | -0.429 | -0.105 | 0.367 | -0.071 | -0.376 | -0.152 | -0.285 | 0.277 | 0.297 | -0.029 | -0.153 |
| VM+VL/*VM+VL* | -0.463 | -0.481 | -0.134 | 0.483 | -0.107 | -0.235 | -0.186 | -0.114 | 0.187 | 0.139 | -0.239 | -0.219 |
| Late stance phase | | | | | | | | | | | | |
| LH/VL | -0.325 | -0.266 | -0.276 | 0.353 | -0.162 | -0.031 | -0.135 | -0.179 | 0.158 | 0.172 | -0.095 | -0.277 |
| MH/VM | -0.311 | -0.232 | -0.184 | 0.195 | -0.175 | -0.142 | -0.138 | -0.290 | 0.297 | 0.121 | -0.118 | -0.168 |
| LH/MH | -0.330 | -0.101 | -0.259 | 0.273 | -0.218 | -0.193 | -0.111 | -0.267 | 0.151 | 0.278 | -0.129 | -0.199 |
| VM/VL | -0.044 | -0.220 | -0.147 | 0.18 | -0.166 | -0.086 | -0.066 | -0.148 | 0.216 | 0.156 | -0.109 | -0.203 |
| LH+MH/*LH+MH* | -0.404 | -0.119 | -0.216 | 0.370 | -0.174 | -0.251 | -0.157 | -0.326 | 0.253 | 0.064 | -0.252 | -0.328 |
| VM+VL/*VM+VL* | -0.366 | -0.429 | -0.204 | 0.372 | -0.116 | - 0.310 | -0.083 | -0.291 | 0.261 | 0.128 | -0.280 | -0.237 |
| Late swing phase | | | | | | | | | | | | |
| LH/VL | -0.209 | -0.153 | -0.298 | 0.207 | -0.084 | -0.316 | -0.034 | -0.185 | 0.206 | 0.227 | -0.321 | -0.147 |
| MH/VM | 0.233 | 0.234 | 0.292 | 0.165 | 0.028 | 0.169 | 0.155 | 0.186 | 0.130 | 0.160 | 0.287 | 0.302 |
| LH/MH | -0.293 | -0.132 | -0.288 | 0.220 | -0.161 | -0.309 | -0.092 | -0.218 | 0.323 | 0.285 | -0.159 | -0.281 |
| VM/VL | 0.239 | 0.275 | 0.289 | 0.158 | 0.181 | 0.317 | 0.156 | 0.154 | 0.281 | 0.022 | 0.271 | 0.171 |
| LH+MH/*LH+MH* | -0.264 | -0.183 | -0.365 | 0.102 | -0.125 | -0.305 | -0.192 | -0.203 | 0.301 | 0.192 | -0.378 | -0.145 |
| VM+VL/*VM+VL* | -0.205 | -0.266 | -0.551 | 0.118 | -0.104 | -0.386 | -0.215 | -0.132 | 0.238 | 0.228 | -0.301 | -0.255 |

*Indicates a significant correlation at P < 0.05

Table S4. Correlation analysis of isokinetic muscle strength parameters and sEMG parameters on the affected side

| Parameters | APTe | PTAe | TPTe | APe | APTf | PTAf | TPTf | APf | H/Q |
| --- | --- | --- | --- | --- | --- | --- | --- | --- | --- |
| VM/VLe | 0.325 | 0.216 | -0.197 | 0.156 | 0.240 | -0.148 | -0.198 | 0.278 | -0.056 |
| MH/LHe | 0.022 | 0.141 | -0.165 | 0.068 | 0.104 | -0.224 | -0.102 | 0.197 | -0.075 |
| MH+LH/VM+VLe | -0.575 | -0.388 | 0.319 | -0.119 | -0.166 | 0.091 | 0.126 | -0.109 | 0.360 |
| MH/*MHe* | - 0.494 | -0.162 | 0.174 | -0.200 | -0.031 | 0.347 | 0.212 | -0.287 | 0.299 |
| LH/*LHe* | -0.539 | -0.392 | 0.399 | -0.087 | -0.308 | 0.013 | 0.089 | -0.261 | 0.230 |
| VM/VLf | 0.266 | 0.262 | -0.325 | 0.104 | 0.461 | -0.061 | -0.082 | 0.296 | -0.226 |
| MH/LHf | 0.092 | 0.086 | -0.183 | 0.118 | 0.093 | -0.227 | -0.071 | 0.155 | -0.283 |
| VM+VL/MH+LHf | -0.025 | -0.065 | 0.074 | -0.118 | -0.482 | 0.430 | 0.310 | -0.146 | 0.406 |
| VM/*VMf* | -0.214 | -0.203 | 0.014 | -0.104 | -0.046 | 0.186 | 0.174 | -0.207 | 0.227 |
| VL/*VLf* | -0.053 | -0.086 | 0.188 | -0.137 | -0.484 | 0.454 | 0.324 | -0.254 | 0.294 |

e and f respectively represent the extension and flexion phases of the knee during the isokinetic muscle strength test. *Indicates a significant correlation at P < 0.05

Table S5. Multivariate regression analysis between gait, muscle strength and muscle activation

| Parameters | *B* | *β* | *P* value | 95% CI |
| --- | --- | --- | --- | --- |
| Speed regression model | | | |  |
| VM+VL/*VM+VL* **-** Mid-stance | -1.787 | -0.984 | 0.023 | -2.837，-0.737 |
| LH+MH/*LH+MH* **-** Late stance | -1.652 | -0.947 | 0.036 | -2.716，-0.588 |
| VM+VL/*VM+VL -* Late stance | -1.330 | -0.653 | 0.010 | -2.293，-0.367 |
| Cadence regression model |  |  |  |  |
| LH+MH/*LH+MH -* Mid-stance | -14.896 | -0.144 | 0.007 | -21.940，-7.852 |
| VM+VL/*VM+VL* - Mid-stance | -35.249 | -0.422 | 0.026 | -54.626，-15.872 |
| VM+VL/*VM+VL* - Late stance | -23.645 | -0.252 | 0.005 | -36.867，-10.423 |
| LH+MH/*LH+MH -* Late swing | -0.857 | -0.797 | 0.039 | -1.478，-0.236 |
| VM+VL/*VM+VL* - Late swing | -0.651 | -0.909 | 0.025 | -1.104，-0.198 |
| Stance time regression model | | | | |
| LH+MH/*LH+MH* - Mid-stance | 5.351 | 0.101 | 0.027 | 1.543，9.159 |
| VM+VL/*VM+VL* - Mid-stance | 6.604 | 0.155 | 0.003 | 3.580，9.628 |
| LH+MH/*LH+MH* - Late stance | 8.823 | 0.215 | 0.035 | 3.097，14.549 |
| VM+VL/*VM+VL* - Late stance | 6.067 | 0.127 | 0.005 | 2.829，9.305 |
| ROM-knee regression model | | | | |
| LH+MH/*LH+MH -* Mid-stance | -27.336 | -0.200 | 0.012 | -43.451，-11.221 |
| VM+VL/*VM+VL -* Late swing | -30.762 | -0.381 | 0.005 | -46.305，-15.219 |
| Peak KFM regression model | | | | |
| LH+MH/*LH+MH -* Early stance | -6.531 | -0.963 | 0.038 | -11.254，-1.808 |
| VM+VL/*VM+VL -* Early stance | -4.115 | -0.665 | 0.028 | -7.078，-1.152 |
| Peak KAM regression model | | | | |
| LH/VL - Early stance | 5.033 | 0.987 | 0.004 | 2.302，7.764 |
| LH/MH - Early stance | 2.052 | 0.919 | 0.013 | 0.807，3.297 |
| LH+MH/*LH+MH* - Early stance | 1.236 | 0.178 | 0.018 | 0.426，2.046 |
| KAM impulse regression model | | | | |
| LH/VL - Early stance | 2.615 | 0.962 | 0.015 | 0.912，4.318 |
| LH/MH - Early stance | 1.174 | 0.987 | 0.012 | 0.300，2.048 |
| LH+MH/*LH+MH* - Early stance | 1.607 | 0.434 | 0.008 | 0.783，2.431 |
| MoS-AP regression model | | | | |
| LH+MH/*LH+MH -* Late swing | -0.528 | -0.810 | 0.015 | -0.900，-0.156 |
| APTe regression model | | | | |
| MH+LH/VM+VLe | -5.308 | -1.087 | 0.013 | -8.953，-1.663 |
| APTf regression model | | | | |
| VM/VLf | 1.217 | 0.889 | 0.038 | 0.341，2.093 |
| VM+VL/MH+LHf | -1.259 | -0.782 | 0.020 | -2.394，-0.124 |
| VL/*VL*f | -1.143 | -0.521 | 0.047 | -2.252，-0.034 |
| H/Q regression model | | | | |
| MH+LH/VM+VLe | 23.053 | 0.206 | 0.022 | 4.746，41.360 |
| VM+VL/MH+LHf | 30.471 | 0.561 | 0.031 | 8.106，52.836 |

e and f respectively represent the extension and flexion phases of the knee during the isokinetic muscle strength test. CI, confidence interva

Table S6. Biomechanical parameters (Continued)

| Knee OA group (n=165) | | | | | | | | | Control(n=32) | p1 | p2 |
| --- | --- | --- | --- | --- | --- | --- | --- | --- | --- | --- | --- |
|  | K-L Ⅰ (n=39) | | K-L Ⅱ (n=38) | | K-L Ⅲ (n=41) | | K-L Ⅳ (n=47) | |  |  |  |
|  | Affected side | Contralateral side | Affected side | Contralateral side | Affected side | Contralateral side | Affected side | Contralateral side |  |  |  |
| Stride length (m)  Male  Female | 1.19±0.15^ɑ^  1.01±0.16* | | 1.11±0.13^ɑ^*  0.91±0.16* | | 1.00±0.15^ɑ^*  0.87±0.19* | | 0.86±0.17^ɑ^*  0.70±0.20* | | 1.29±0.11^ɑ^  1.20±0.13 | <0.001* | <0.001* |
| ROM-hip (°)  Male  Female | 42.08±9.27  41.93±8.66 | 43.91±9.31  41.22±7.86 | 39.71±10.51  38.82±9.87 | 40.21±7.23  39.63±8.21 | 35.84±11.22*  33.27±9.86* | 38.71±8.26  37.51±8.57 | 31.66±10.71*  27.54±12.24* | 35.29±9.07  33.39±10.05 | 44.01±10.33  43.21±11.76 | <0.001* | <0.001* |
| ASI_1_/ASI_2_ | 4.25±3.37/4.06±3.23 | | 4.60±3.58/4.13±3.22 | | 4.87±3.70/4.53±3.47 | | 5.42±3.93/5.12±3.70 | |  |  |  |
| ROM-ankle (°)  Male  Female | 32.61±5.25  32.07±6.40 | 33.26±5.13  34.15±6.72 | 30.38±7.16  29.11±8.09 | 32.94±6.91  30.72±5.24 | 26.05±9.49  24.12±11.36* | 29.13±9.41  26.85±8.77 | 21.75±11.29*  20.29±13.21* | 27.81±8.27  24.77±7.43 | 33.64±6.04  32.81±7.11 | <0.001* | <0.001* |
| ASI_1_/ASI_2_ | 4.04±3.17/3.92±3.05 | | 4.17±3.25/3.79±2.95 | | 4.57±3.44/4.14±3.13 | | 5.30±3.73/4.82±3.44 | |  |  |  |
| KAM impulse (Nm.s/(BW×HT)%)  Male  Female | 1.02±0.60  1.17±0.66 | 1.06±0.43  1.19±0.54 | 1.19±0.65  1.25±0.72 | 1.22±0.45  1.31±0.56 | 1.43±0.67*  1.52±0.75* | 1.55±0.61  1.60±0.64 | 1.62±0.89*  1.86±0.96* | 1.71±0.88  1.93±0.85 | 0.87±0.40  1.10±0.58 | 0.007* | <0.001* |
| ASI_1_/ASI_2_ | 5.24±4.06/4.85±3.68 | | 4.98±3.78/4.59±3.41 | | 5.52±4.16/5.17±3.83 | | 5.95±4.49/5.81±4.26 | |  |  |  |
| TPT (s)  Male  Female | 0.73±0.15  0.78±0.19 | 0.71±0.11  0.79±0.12 | 0.80±0.20  0.87±0.19 | 0.75±0.15  0.81±0.13 | 0.90±0.28  0.92±0.25* | 0.77±0.11  0.81±0.17 | 0.99±0.35*  1.01±0.31* | 0.82±0.15  0.83±0.11 | 0.70±0.13  0.77±0.10 | 0.002* | 0.026* |
| LSI_1_/LSI_2_ (%) | 92.7 ± 5.6/90.6 ± 5.4 | | 83.3 ± 6.2/80.7 ± 6.5 | | 72.7 ± 6.5/69.8 ± 7.0 | | 60.9 ± 5.5/58.7 ± 5.9 | |  |  |  |
| AP (w)  Male  Female | 91.85±14.10^ɑ^  81.43±13.71* | 96.97±10.05  89.29±9.63 | 80.11±16.80^ɑ^*  69.33±12.60* | 90.55±9.28  87.35±8.86 | 65.03±18.57*  56.42±17.56* | 85.76±12.16  84.52±11.35 | 49.41±18.64*  38.82±16.94* | 80.68±13.53  77.23±11.31 | 98.57±16.34  89.71±12.26 | <0.001* | <0.001* |
| LSI_1_/LSI_2_ (%) | 91.8 ± 5.5/89.5 ± 5.4 | | 82.4 ± 6.2/79.6 ± 6.4 | | 71.8 ± 6.5/68.7 ± 6.9 | | 60.1 ± 5.4/57.9 ± 5.8 | |  |  |  |
| TPT (s)  Male  Female | 0.59±0.15  0.62±0.19 | 0.62±0.15  0.60±0.17 | 0.64±0.22  0.68±0.27 | 0.63±0.16  0.62±0.20 | 0.74±0.33  0.77±0.28 | 0.66±0.19  0.64±0.24 | 0.83±0.30*  0.85±0.25* | 0.69±0.25  0.69±0.18 | 0.60±0.17  0.59±0.13 | 0.004* | <0.001* |
| LSI_1_/LSI_2_ (%) | 91.2 ± 5.5/88.7 ± 5.3 | | 81.6 ± 6.1/78.7 ± 6.3 | | 70.9 ± 6.5/67.8 ± 6.8 | | 59.1 ± 5.3/56.8 ± 5.7 | |  |  |  |
| AP (w)  Male  Female | 61.14±14.05  54.39±13.22 | 63.68±14.77  59.72±10.38 | 53.68±19.01  45.78±16.73* | 58.88±13.86  61.36±12.17 | 44.32±18.36  40.99±17.87* | 57.19±16.17  63.05±16.96 | 35.12±19.34*  28.83±13.27* | 54.87±13.03  57.33±18.73 | 62.14±13.74  60.42±15.32 | <0.001* | <0.001* |
| LSI_1_/LSI_2_ (%) | 91.5 ± 5.5/89.0 ± 5.3 | | 82.1 ± 6.2/79.2 ± 6.3 | | 71.4 ± 6.5/68.3 ± 6.8 | | 59.6 ± 5.4/57.3 ± 5.7 | |  |  |  |

Data are presented as mean±standard deviation. K-L, Kellgren-Lawrence grade. RoM, range of motion in the sagittal plane. KAM, knee adduction moment. TPT, time to peak torque. AP, average power. ASI, Asymmetry index. ASI_1_ and ASI_2_ represent the lower limb symmetry of males and females, respectively. ASI, Asymmetry index. ASI_1_ and ASI_2_ represent the limb symmetry of males and females, respectively. An ASI value of "0" indicates absolute symmetry between the two lower limbs, while a larger ASI value indicates a higher degree of asymmetry. LSI, limb symmetry index. LSI_1_ and LSI_2_ represent the limb symmetry of males and females, respectively. Statistical value refers to ANOVA or the independent Student's t-test. The overall ANOVA p-values (p1 for males, p2 for females) indicate whether significant differences exist among the five groups. * indicates significant difference between KOA groups and control group after Bonferroni correction, ɑ indicates significant difference between males and females within the same K-L grade (independent t-test, p < 0.05).
